# Supplementary material for: Effects of Ingesting Fucoidan Derived from Cladosiphon okamuranus Tokida on Human NK Cells: A Randomized, Double-Blind, Parallel-Group, Placebo-Controlled Pilot Study
Source: Mar Drugs. 2021 Jun 15;19(6):340. doi: 10.3390/md19060340 (PMC8232719; doi:10.3390/md19060340)
Supplement: Supplementary file 1 [file marinedrugs-19-00340-s001.zip › marinedrugs-1217213-supplementary.pdf]

Supplementary Materials

Effects of ingesting fucoidan derived from Cladosiphon okamuranus Tokida on human NK cells: a randomized, double-blind, parallel-group, placebo-controlled pilot study.

Makoto Tomori, Takeaki Nagamine, Tomofumi Miyamoto, and Masahiko Iha

NK cell activity

Table S1. NK cell activity in fucoidan group

No	Gender	Age	0W	4W	8W	12W	16W
1	female	48	36.8	53.7	70.9	56.5	54.7
2	female	60	80.9	74.4	68.4	75.5	67.2
3	male	49	72.3	89.9	85.9	89.4	79.4
4	female	44	36.0	31.9	39.6	34.6	35.4
5	male	45	57.9	59.1	61.5	64.8	35.3
6	male	34	73.3	86.5	83.9	83.6	74.7
7	male	49	25.8	63.4	64.8	71.3	52.0
8	male	37	77.3	85.2	78.7	87.2	75.1
9	female	43	60.7	65.4	69.6	67.3	46.0
10	male	42	68.9	65.9			
11	male	59	86.0	88.7	83.8	86.2	74.9
12	male	32	30.6	44.1	58.9	42.0	36.4
13	female	55	82.0	86.9	80.1	89.8	79.9
14	female	41	64.5	65.1	63.4	59.2	42.3
15	female	54	64.1	68.7	64.8	67.2	58.3
16	male	46	45.2	57.2	52.7	42.6	49.2
17	female	41	31.1	38.8	39.8	40.5	44.9
18	female	40	49.4	38.3	62.6	61.4	56.8
19	male	52	73.9	87.9	77.9	85.3	80.0
20	female	39	69.8	84.1	80.9	75.1	61.8

Unit is percentage(%).
Normal range: 1) male: 44.1-75.4%, 2) female: 36.7-63.4%

Table S2. NK cell activity in placebo group

No	Gender	Age	0W	4W	8W	12W	16W
1	female	61	50.6	59.2	67.0	80.7	69.5
2	female	57	30.7	47.2	38.4	48.0	45.2
3	male	49	81.5	81.6	81.2	62.4	77.2
4	female	37	40.2	46.9	53.0	11.3	41.7
5	male	36	86.2	89.3	82.5	84.8	86.3
6	male	44	54.7	75.4	58.1	76.8	69.1
7	male	49	75.1	70.7	64.2	80.3	58.4
8	female	46	61.7	80.4	71.9	73.9	63.7
9	male	54	64.1	74.9	56.2	63.2	55.3
10	female	52	68.6	81.3	75.7	78.5	67.0
11	female	43	55.9	60.7	64.3	70.3	48.3
12	male	40	58.0	50.0	50.4	59.5	53.9
13	female	48	67.4	63.1	61.9	64.7	57.9
14	female	42	50.1	53.4	41.6	32.1	51.5
15	male	46	63.8	63.5	58.6	65.6	47.8
16	female	39	68.1	64.0	63.7	78.9	50.5
17	male	62	67.6	69.8	59.6	68.7	61.5
18	male	45	53.6	63.2	42.0	64.2	55.1
19	female	38	57.5	57.8	55.2	58.9	56.2
20	male	51	75.1	76.9	73.6	83.5	75.5

Unit is percentage(%).
Normal range: 1) male: 44.1-75.4%, 2) female: 36.7-63.4%

IFN-γ

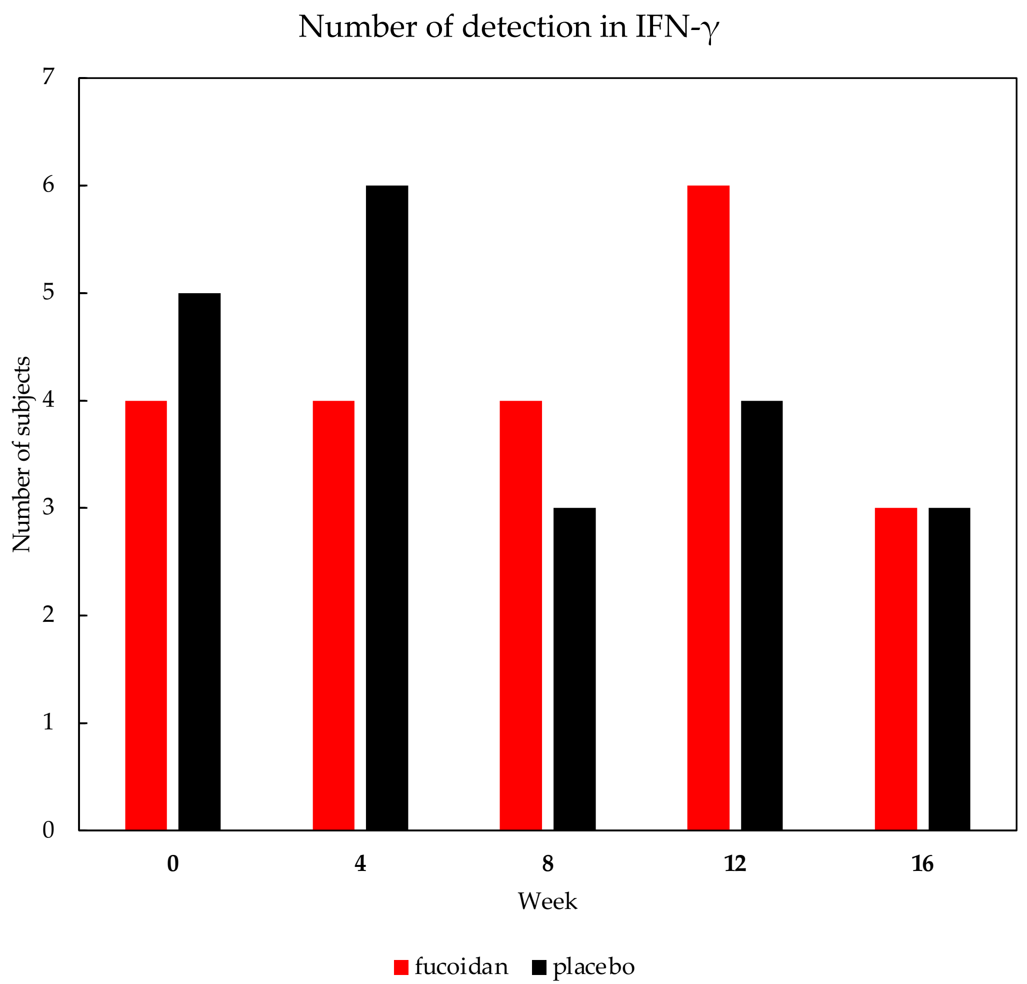

Figure S1. between-group comparison of subjects in which IFN-γ concentrations were detected in the blood. Detection limit: >1.56 pg/mL.

Blood test

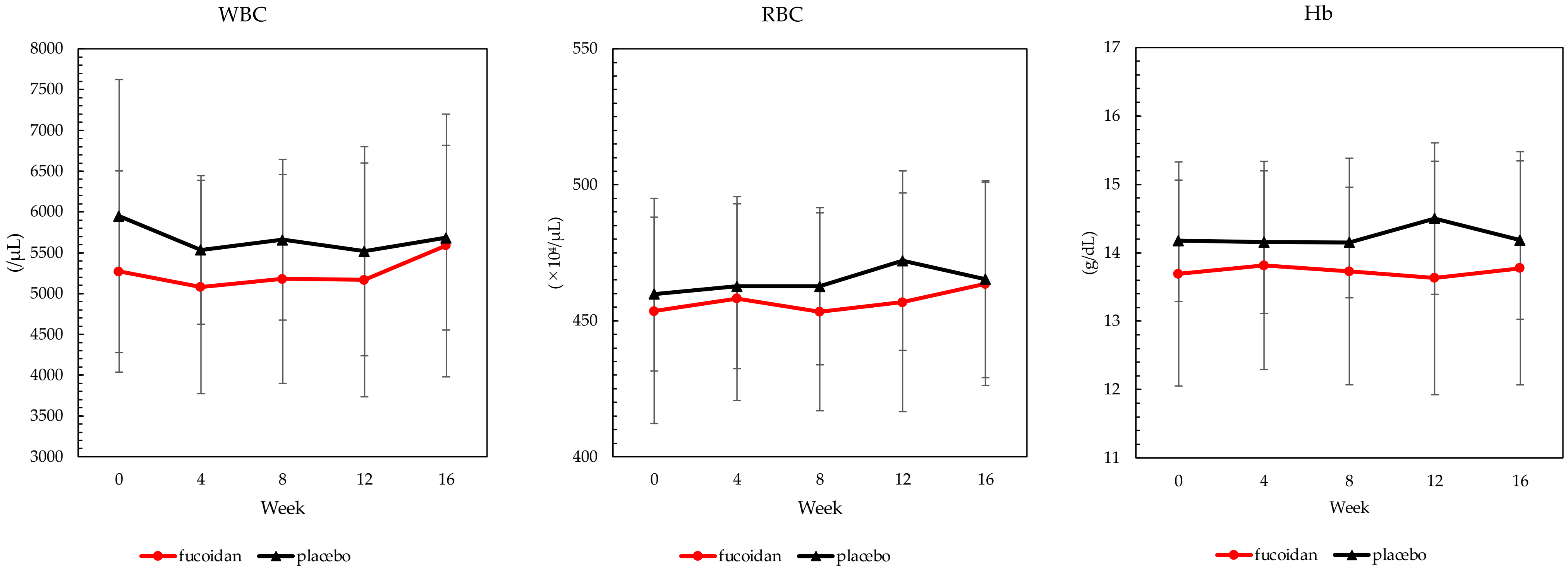

Figure S2. Blood test result. all data was presented as mean ± standard deviation. WBC, RBC and Hb were no significant different.

Blood test

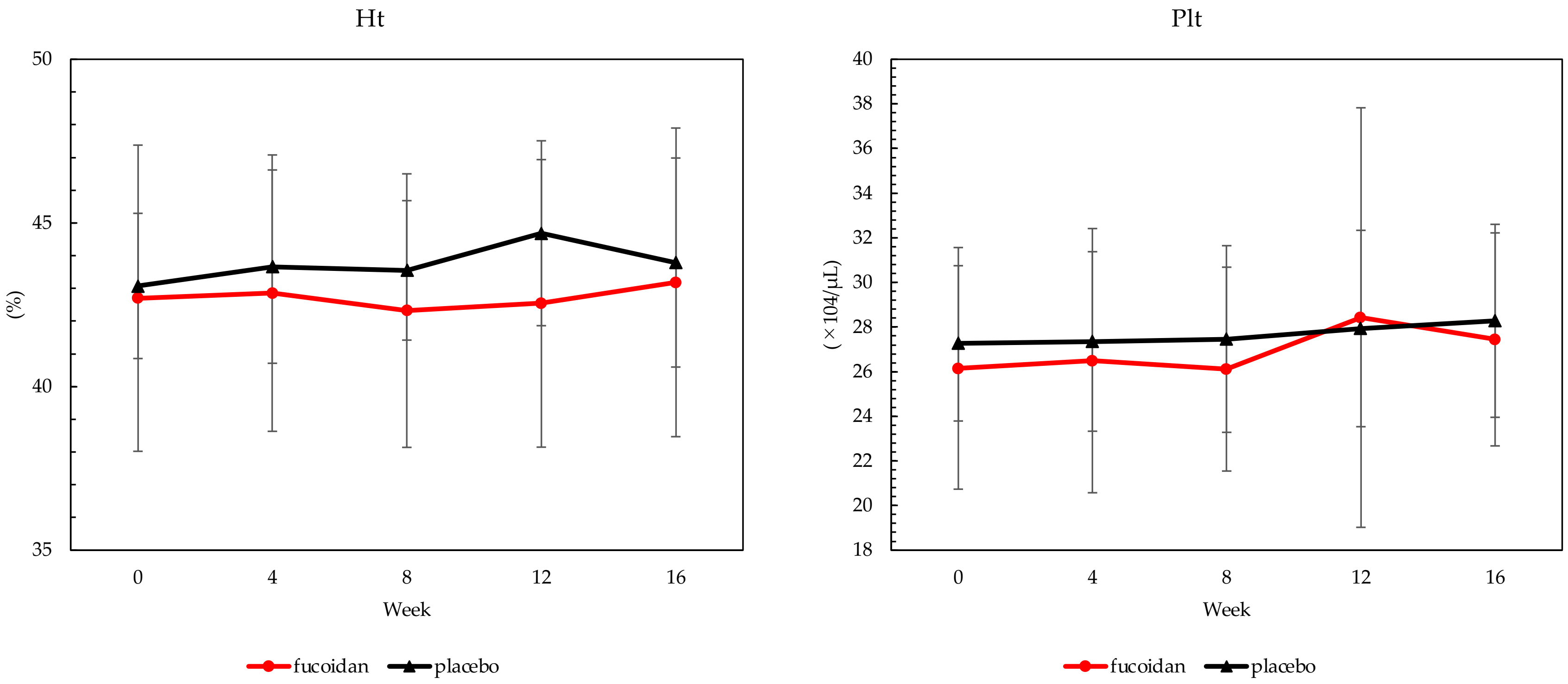

Figure S3. Blood test result. all data was presented as mean ± standard deviation. Ht and Plt were no significant different.

Biochemical test

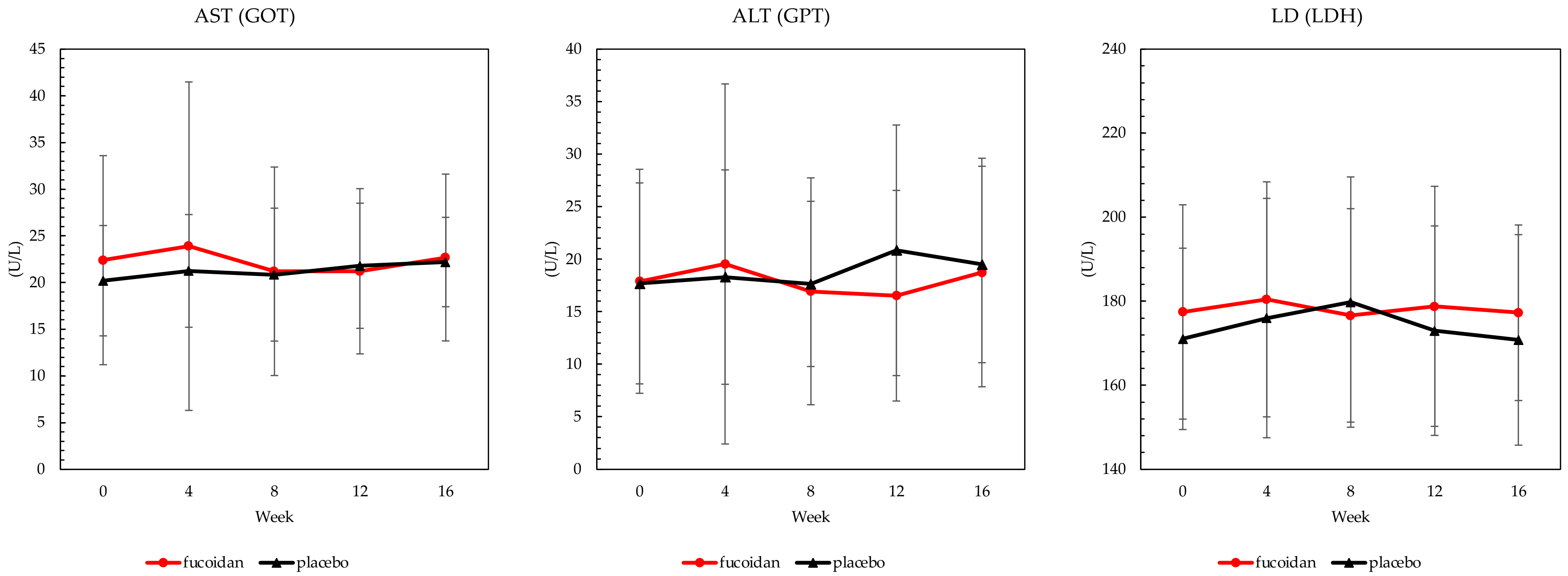

Figure S4. Biochemical test result. all data was presented as mean ± standard deviation.
AST, ALT and LF were no significant different.

Biochemical test

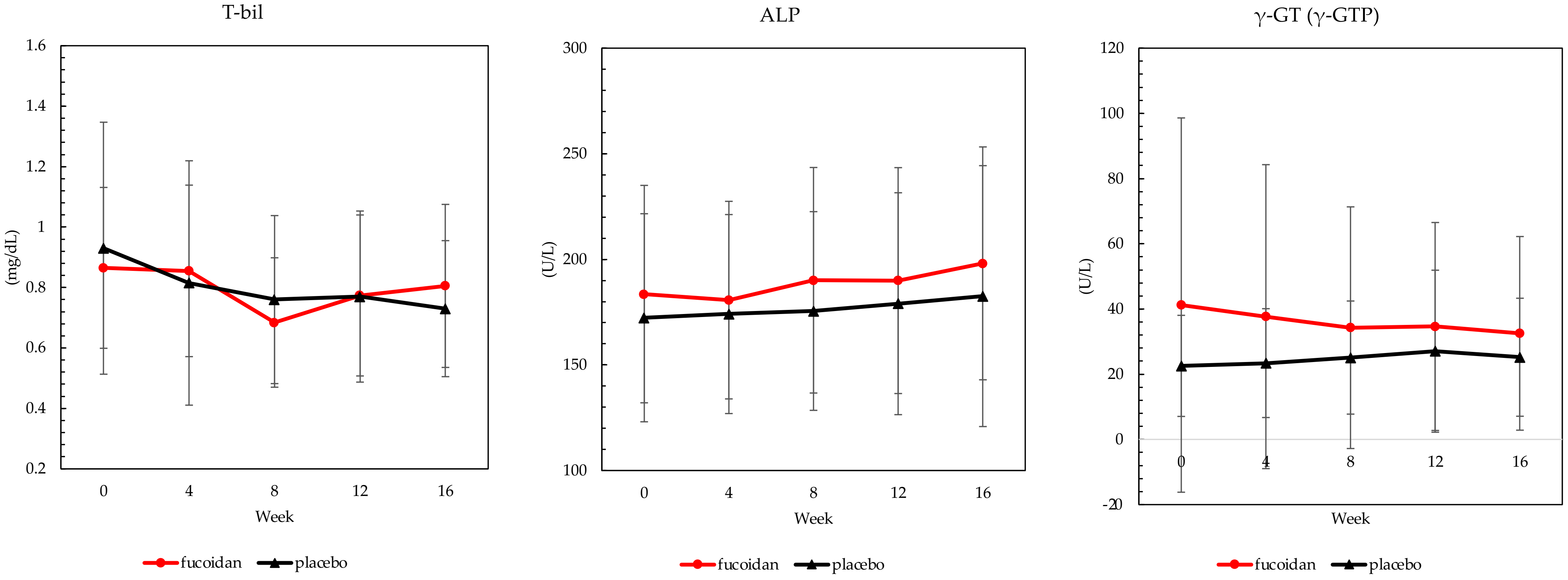

Figure S5. Biochemical test result. all data was presented as mean ± standard deviation.
T-bill, ALP and γ-GT were no significant different.

Biochemical test

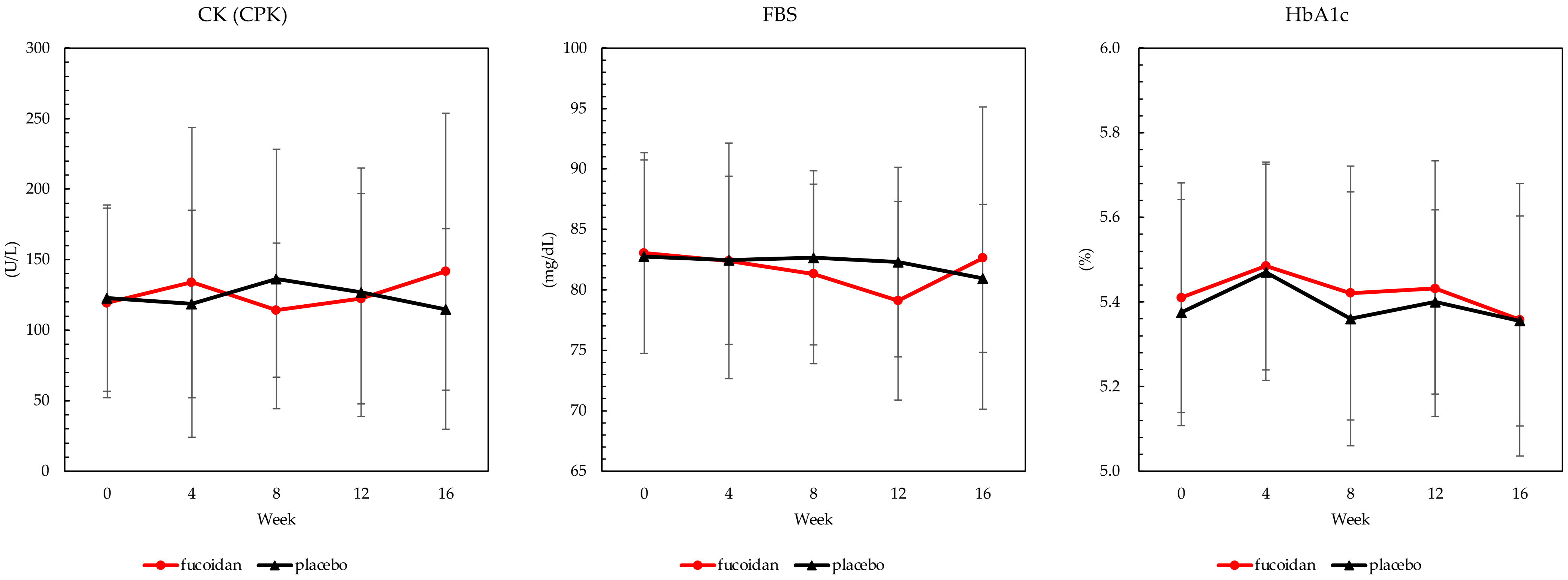

Figure S6. Biochemical test result. all data was presented as mean ± standard deviation.
CK, FBS and HbA1c were no significant different.

Biochemical test

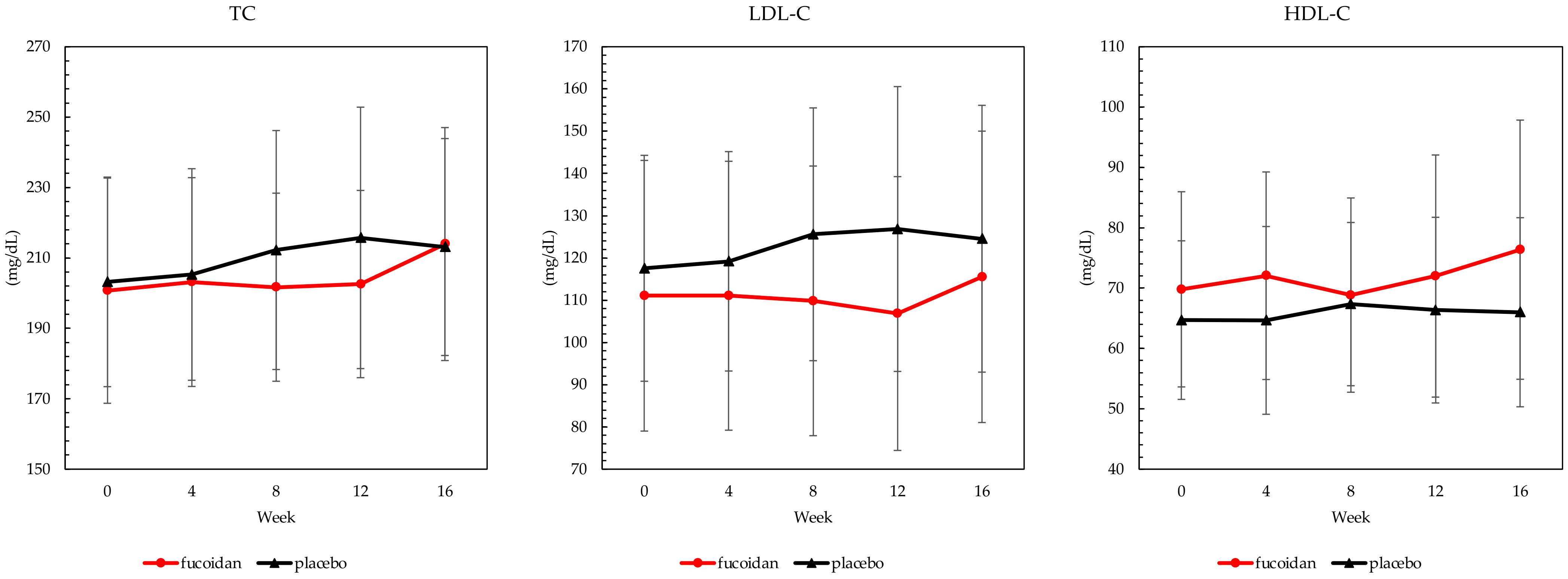

Figure S7. Biochemical test result. all data was presented as mean ± standard deviation.
TC, LDL-C and HDL-C were no significant different.

Biochemical test

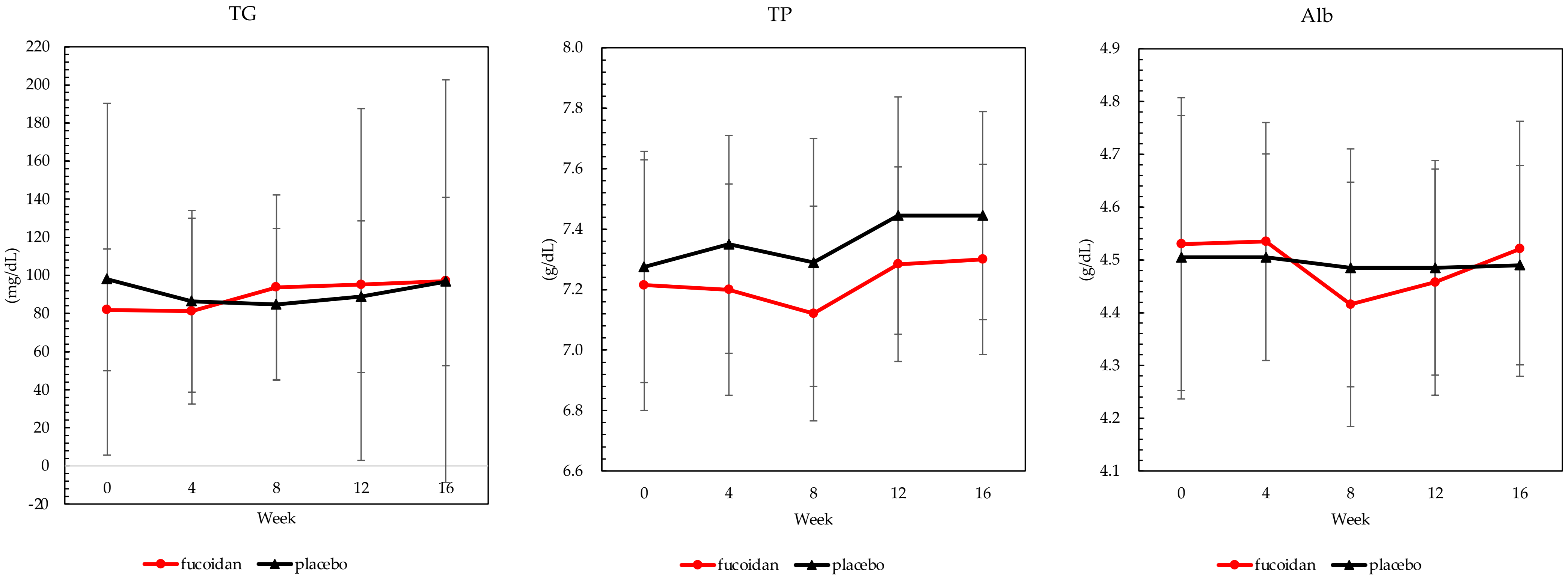

Figure S8. Biochemical test result. all data was presented as mean ± standard deviaion.
TG, TP and Alb were no significant different.

Biochemical test

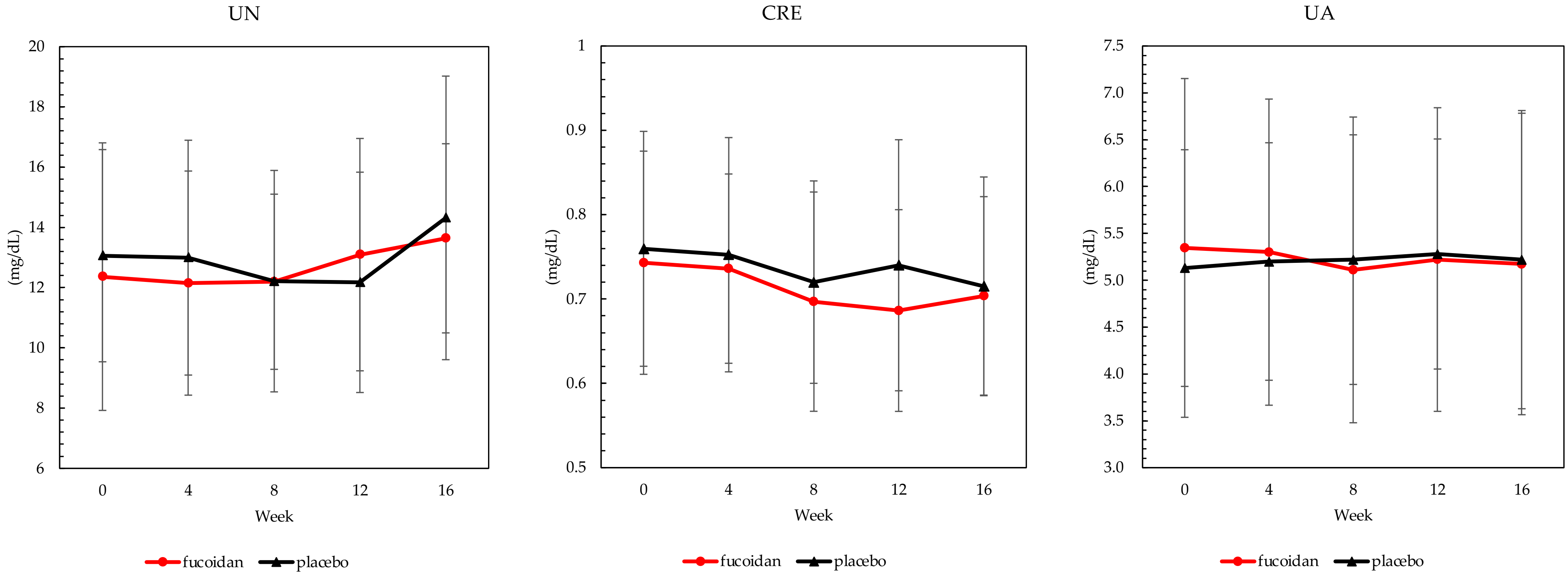

Figure S9. Biochemical test result. all data was presented as mean ± standard deviation.
UN,CRE and UA were no significant different.

Biochemical test

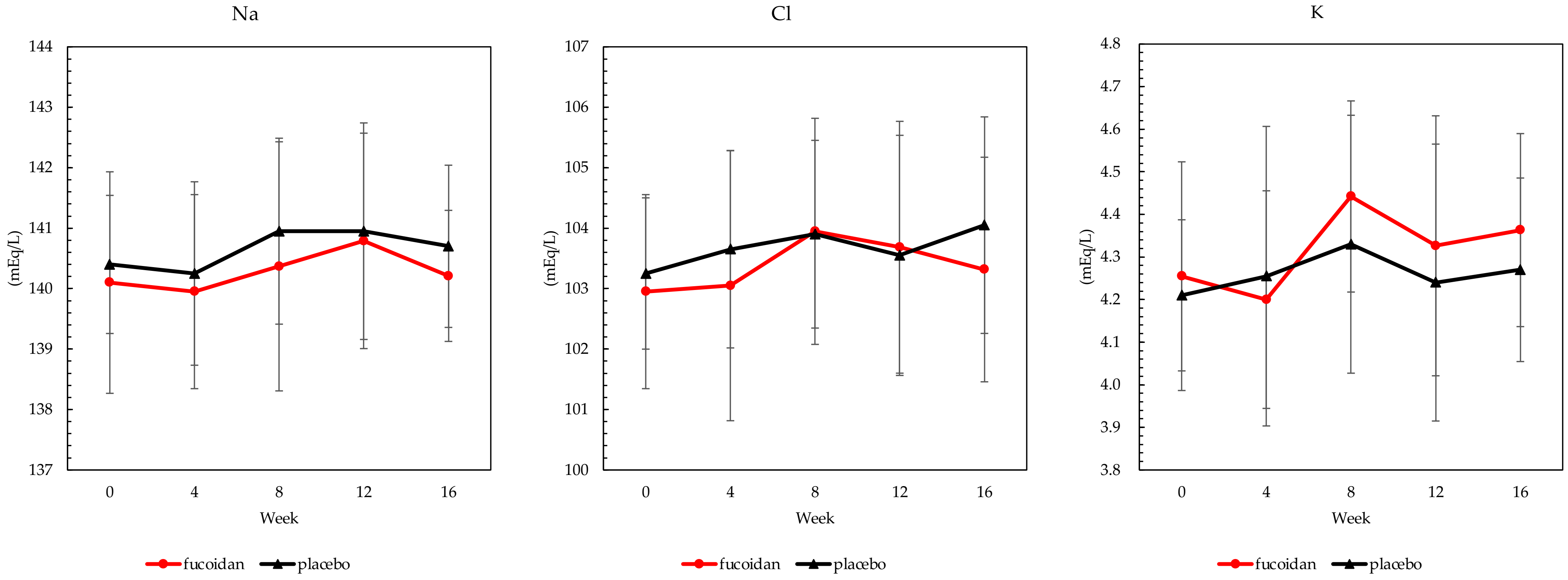

Figure S10. Biochemical test result. all data was presented as mean ± standard deviation.
Na, Cl and K were no significant different.

Biochemical test

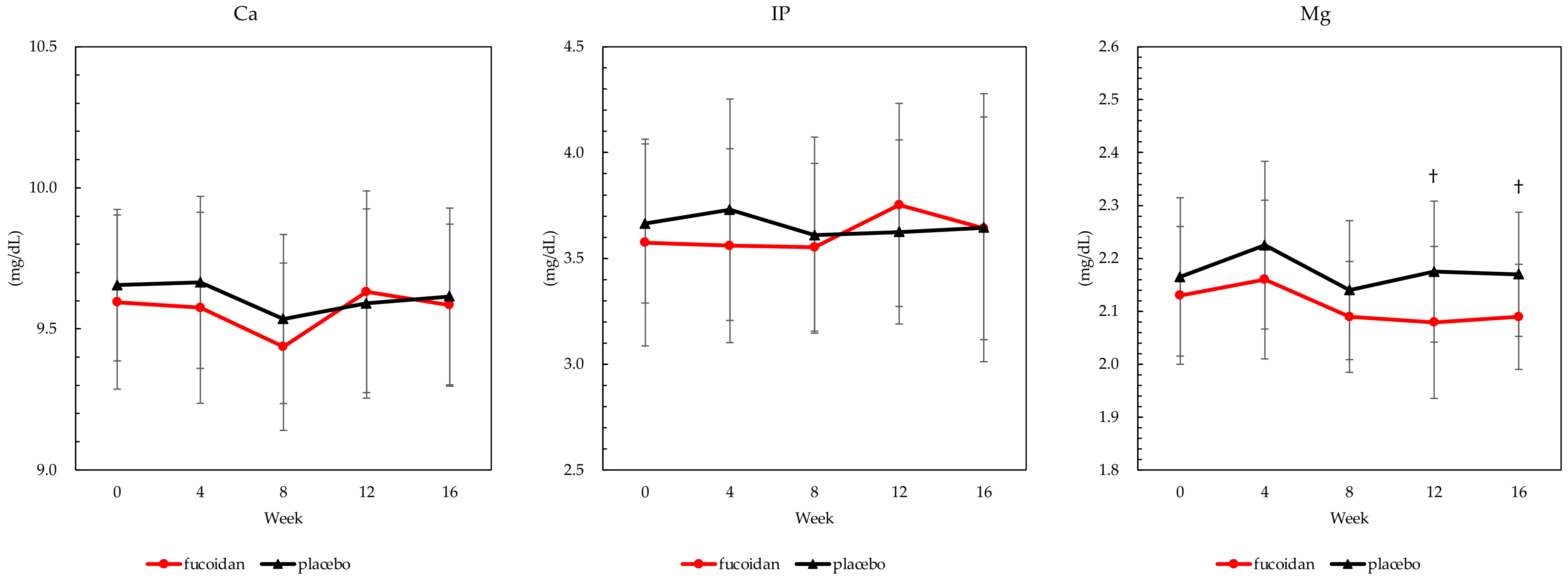

Figure S11. Biochemical test result. all data was presented as mean ± standard deviation.
Ca and IP were no significant different.
†There was a significant difference between the 12 w and 16 w of the fucoidan and placebo group. ($p<0.05$, t-test).

Biochemical test

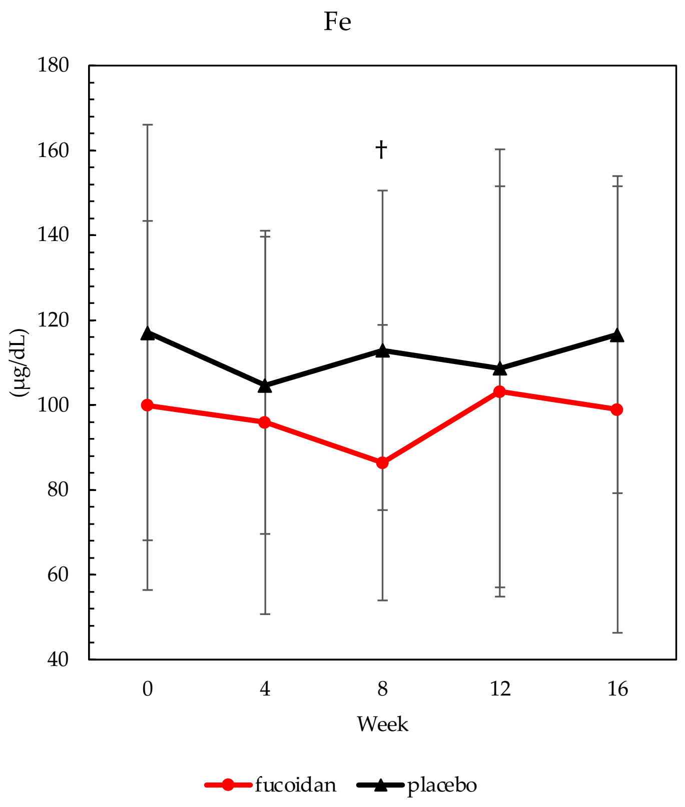

Figure S12. Biochemical test result. all data was presented as mean ± standard deviation.
†There was a significant difference between the 8 w of the fucoidan and placebo group. ($p<0.05$, t-test).
